# Emerging role of RNA modification and long noncoding RNA interaction in cancer

**DOI:** 10.1038/s41417-024-00734-2

**Published:** 2024-02-14

**Authors:** Liqiong Yang, Lu Tang, Qi Min, Hua Tian, Linwei Li, Yueshui Zhao, Xu Wu, Mingxing Li, Fukuan Du, Yu Chen, Wanping Li, Xiaobing Li, Meijuan Chen, Li Gu, Yuhong Sun, Zhangang Xiao, Jing Shen

**Affiliations:** 1 Laboratory of Molecular Pharmacology, Department of Pharmacology, School of Pharmacy, Southwest Medical University, Luzhou, 646000 China; 2 Cell Therapy & Cell Drugs of Luzhou Key Laboratory, Luzhou, 646000 China; 3 South Sichuan Institute of Translational Medicine, Luzhou, 646000 China; 4 Department of Oncology, The Affiliated Hospital of Southwest Medical University, Luzhou, 646000 China

**Keywords:** Cancer, Gene regulation

## Abstract

RNA modification, especially N6-methyladenosine, 5-methylcytosine, and N7-methylguanosine methylation, participates in the occurrence and progression of cancer through multiple pathways. The function and expression of these epigenetic regulators have gradually become a hot topic in cancer research. Mutation and regulation of noncoding RNA, especially lncRNA, play a major role in cancer. Generally, lncRNAs exert tumor-suppressive or oncogenic functions and its dysregulation can promote tumor occurrence and metastasis. In this review, we summarize N6-methyladenosine, 5-methylcytosine, and N7-methylguanosine modifications in lncRNAs. Furthermore, we discuss the relationship between epigenetic RNA modification and lncRNA interaction and cancer progression in various cancers. Therefore, this review gives a comprehensive understanding of the mechanisms by which RNA modification affects the progression of various cancers by regulating lncRNAs, which may shed new light on cancer research and provide new insights into cancer therapy.

## Introduction

In recent years, more and more researchers have focused on non-protein-coding genomes. 70% of the transcribed human genome corresponds to noncoding RNAs (ncRNA) [1]. Long noncoding RNAs (lncRNAs) are a group of ncRNAs over 200 nucleotides in length. The structures of lncRNA are complex and diverse, including linear, circular, Y-shaped, U-shaped, and other shapes. In addition, lncRNAs tend to fold into complex secondary and tertiary structures and interact with proteins, DNA, and other RNAs, regulating the activity of multi-protein complexes and DNA targets. These special structures can not only affect the function of lncRNA, but also affect their stability and interaction [2]. The functions of lncRNA are highly diversified. For example, lncRNA can regulate gene transcription by acting as decoys or guiding chromatin modifiers. They can also function as recruiters and scaffolds for other regulatory factors involved in epigenetic modifications. In addition, they are also implicated in mRNA processing, splicing, stability, or translation. In the cytoplasm, lncRNAs may act as scaffolds to bring two or more proteins into spatial proximity to each other [3, 4]. Therefore, lncRNAs can execute a variety of molecular functions, including epigenetic regulation, transcriptional regulation, post-transcriptional regulation, miRNA regulation, and regulating the activity of proteins or altering their cellular localization [5]. LncRNAs can also participate in multiple signal pathways of cancer (such as p53, AKT, or Notch), epigenetic control, DNA damage, multiple biological functions (e.g., tumor proliferation, metabolism, and apoptosis, etc.), aerobic glycolysis, and microRNA control, etc., suggesting that they are important players in cancer [6–14]. Aberrant expression of lncRNAs can affect the occurrence, progress, and drug resistance of cancer [15]. In recent years, many reviews have summarized the role of lnRNA in cancer [16, 17]. For example, lncRNA HULC promotes breast cancer metastasis and cisplatin resistance by targeting the IGF1R-PI3K-AKT axis in trans [18]. Therefore, the multifaceted functions of lncRNAs make them potential therapeutic targets or biomarkers in various cancers.

Epigenetics refers to the change of gene expression level without changing gene sequence, which includes DNA methylation, chromosome remodeling, protein post-translational modification, histone modification, and ncRNA regulation [19]. In animal cells, proteins and RNAs have the greatest variety of modifications, and researchers have discovered a variety of modifications over the past 50 years [20]. Modification of proteins has been extensively characterized. Research on RNA modification has also increased in recent years. The disorder of RNA epigenetic pathway is related to the pathogenesis of human diseases, including cancer. Up to now, more than 170 different types of RNA modifications have been reported to modify different RNA types, including messenger RNA (mRNA), transfer RNA (tRNA), ribosomal RNA (rRNA), and lncRNA [21]. As one of the important epigenetic modifications, RNA methylation is closely related to the occurrence and development of cancer. It is expected to become a new target for cancer treatment. [22, 23]. Among this RNA methylation, the most well-studied one in recent years is N6-methyladenosine (m6A), the nitrogen 6 of adenosine (A) found in mRNA and ncRNA. The m6A methylation can be dynamic and reversible, which is modulated by specific enzymes called “erasers” (demethylases), “readers” (signal transducers), and “writers” (methyltransferases). These modulators of the m6A modification process have been extensively studied and shown to be important players in cancer progression [24]. Moreover, trans regulators of m6A have also been identified to play important roles in cancer development [25, 26]. Additionally, other RNA modifications in mammals include A-to-I RNA editing, 2′-O-methylation (2′-O-Me), N1 -methyladenosine (m1A), 3-methylcytidine (m3C), 5-methylcytosine (m5C), N7-methylguanosine (m7G), pseudouridylation (Ψ), etc, which are all recently discovered epigenetic modifications [27]. Among them, the mechanism of m6A in cancer is the most extensively studied, followed by m5C and m7G [28].

In recent years, the inter-relationship between RNA modification and lncRNA has gradually been discovered. LncRNA can regulate gene expression through RNA modification and exert its biological role. At the same time, lncRNAs are also subjected to RNA modification [29]. Up to date, reported RNA modifications associated with lncRNAs include m6A, m5C, and m7G. Among them, m6A is the most well-investigated. The modification of m6A on lncRNA can increase the stability of lncRNA, thus affecting various biological functions of cancer cells through ceRNA (competing endogenous RNA) mechanism [30]. RNA modification can also affect the lncRNA structure, thereby influencing the regulation of protein by lncRNA. In addition, RNA modification can also promote lncRNA-mediated transcriptional silencing [31]. Finally, RNA modification can change the subcellular distribution of lncRNA and regulate its stability [32–35]. Although some substantial progress has been recently made in the RNA modification mechanisms of ncRNAs, the modification of lncRNAs has not been well elucidated. In this review, we summarize the interaction of RNA modification and lncRNAs and its function in cancer, which may provide new perspectives on lncRNAs in cancer research.

## RNA modification and lncRNA

### m6A modification in lncRNA

**Fig. 1:**
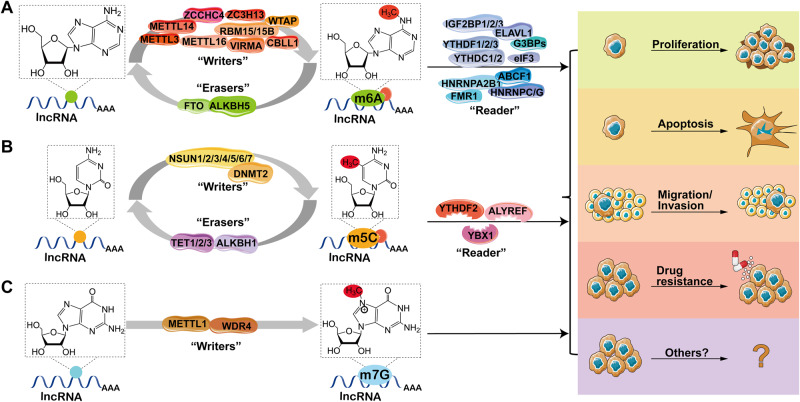
The role of RNA modifications in cancer proliferation, apoptosis, migration and invasion, and drug resistance. **A** m6A in lncRNA. The m6A modification is modulated by m6A “writers”, “erasers”, and “readers”. m6A “writers” are methylase complexes including METTL3, METTL14, METTL16, ZC3H13, ZCCHC4, RBM15/15B, VIRMA, CBLL1, and WTAP. “Erasers” are demethylases (FTO, ALKHB5) that remove methyl groups. The m6A-containing RNAs are recognized by “readers”, including HNRNPA2B1, IGF2BP1/2/3, YTHDC1/2, YTHDF1/2/3, ABCF1, eIF3, FMR1, HNRNPC/G, ELAVL1, and G3BPs. **B** m5C in lncRNA. m5C modification in lncRNA regulated by NSUN family proteins (NSUN1, NSUN2, NSUN3, NSUN4, NSUN5, NSUN6), and DNMT2. And recognized by “readers”, including YTHDF2, ALYREF, and YBX1. “Erasers” include TET1/2/3, ALKBH1, for m5C demethylation. **C** m7G in lncRNA. The METTL1/WDR4 complex is the methylase of m7G. “Eraser” and “reader” are unknown.

m6A is adenosine methylated at the sixth N position, which is the most typical RNA modification (Fig. 1). This modification is found in mRNA [36], lncRNAs [37], primary miRNA (pri-miRNA) [38] and rRNA [39]. It is reported to perform important functions affecting normal life activities and disease. Approximately 25% of mRNAs contain at least one m6A, and mRNAs can contain up to 0.1–0.4% of modified sites [40, 41]. m6A sites occur frequently around stop codons, in the 3′-untranslated region (UTR), and in long exons, with the most common m6A consensus motif: RRACH (R = A or G, H = A, U, or C) [27]. Adenosine is methylated by methyltransferase-like 3/14/16 (METTL3/14/16), Wilm’s tumor-associated protein (WTAP), RNA-binding motif protein 15 (RBM15) and its paralog RBM15B, Vir-like m6A methyltransferase associated (VIRMA, also called KIAA1429), zinc finger CCHC-type containing 4 (ZCCHC4), and zinc finger CCCH-type containing 13 (ZC3H13) [42]. This kind of enzyme is called “m6A writers”. Then these m6A-modified bases are demethylated by AlkB homolog 5 (ALKBH5) and fat mass and obesity-associated protein (FTO) [43, 44], which are called “m6A erasers”. Finally, methylated RNA base sites require specific enzymes to recognize them. These are called “m6A readers”, including IYT521-B homology (YTH) family proteins, heterogeneous nuclear ribonucleoprotein (hnRNP) and eukaryotic translation initiation factor (eIF), etc. The proteins with YTH domain include YTH domain-containing 1/2 (YTHDC1/2) and YTH m6A-binding protein 1/2/3 (YTHDF1/2/3) [45]. Binding of YTHDC1 to m6A regulates splicing, while YTHDF2 targets the transcript for degradation [46–50]. Recruitment of YTHDF1, YTHDF3, and YTHDC2 enhances translation [51, 52]. Insulin-like growth factor 2 mRNA-binding proteins (IGF2BPs) as a new m6A reader protein, also increases the stability of its targeted RNA [53]. Taken together, m6A affects RNA stability, splicing, localization, and translation at the post-transcriptional level, as shown in Table 1.

**Table 1:** The functional roles of m6A, m5C, and m7G regulators in RNA metabolism.

Protein		Function	References
m6A	*Writers*		
	METTL3	Installs m6A residues in mRNAs and lncRNAs	[209]
	METTL14	Activates, and escalates the catalytic capability of METTL3	[210]
	METTL16	Installs m6A in U6 snRNA and few mRNAs and lncRNAs	[211]
	WTAP	Recruits METTL3 and METTL14	[212]
	VIRMA	Regulates m^6^A installation by the METTL3/METTL14 complex	[213]
	RBM15/15B	Recruits METTL3 and METTL14 complex to RNA	[214]
	CBLL1	Maintain the stability of METTL3/14	[213]
	ZC3H13	Recruits m6A complex	[214]
	ZCCHC4	Recruitment of m6A complex to 28S rRNA	[215]
	*Erasers*		
	FTO	Remove m^6^A and m^6^Am from mRNA, and m^1^A from tRNA	[43]
	ALKBH5	Remove m^6^A from mRNA	[44]
	*Readers*		
	HNRNPA2B1	Stimulates microRNA processing	[216]
	IGF2BP1/2/3	Promotes mRNA stability	[53]
	YTHDC1	Regulates splicing	[217]
	YTHDC2	Enhances translation	[218]
	YTHDF1	Promotes translation	[219]
	YTHDF2	Stimulates mRNA decay	[220]
	YTHDF3	Enhances translation	[51]
	ABCF1	Stimulates cap-independent translation	[221]
	eIF3	Binds to the base of m6A modification on the 5′UTRs of RNA and promotes mRNA translation	[222]
	FMR1	Inhibits translation	[223]
	HNRNPC, HNRNPG	Regulates splicing	[224]
	ELAVL1/ HuR	Increases RNA stability	[225]
	G3BPs	Increases mRNA stability	[223]
m5C	*Writer*		
	NSUN1(NOP2)	Methylate C2870 of the 25S rRNA	[226]
	NSUN2	Modify the noncoding Vault RNA and mRNAs	[66]
	NSUN3	Methylate cytosine 34 of mitochondrial tRNA	[227]
	NSUN4	Modify rRNA	[228]
	NSUN5	Modify 28S rRNA	[229]
	NSUN6	Methylate cytosine 72 of cytoplasmic tRNA	[230]
	NSUN7	Targets eRNAs as a substrate	[231]
	DNMT2	Modify cytoplasmic tRNA	[232]
	*Erasers*		
	TET1/2/3	Promotes subsequent oxidation to replace modification	[233]
	ALKBH1	Catalyzes the anticodon modification	[234]
	*Readers*		
	YTHDF2	Binding m5C-modified RNA	[235]
	ALYREF	Enhanced m5C modification and binding ability	[67]
	YBX1	Recognized and bind m5C-modified mRNA via Trp45	[236]
m7G	*Writer*		
	METTL1	Promotes m7G modification	[93]
	WDR4	Strengthening the stability of METTL1 complex	[92]

*CBLL1* Cbl proto-oncogene like 1, *HNRNPA2B1* heterogeneous nuclear ribonucleoprotein A2/B1, *ABCF1* ATP binding cassette subfamily F member 1, *eIF3* eukaryotic translation initiation factor (eIF) 3, *FMR1* fragile X messenger ribonucleoprotein 1, *HNRNPC* heterogeneous nuclear ribonucleoprotein C, *ELAVL1* ELAV like RNA-binding protein 1, *DNMT2* DNA methyltransferase-2.

Most studies suggest that m6A modifications can affect the complexity of cancer progression by modulating cancer-related biological functions [54–56]. m6A modification of mRNA is well documented [57]. Methylated RNA immunoprecipitation and sequencing (MeRIP-Seq) data suggested that m6A modification also exists in lncRNA, albeit in much smaller numbers [58]. Notably, lncRNAs also play important roles in regulating these m6A modifications. m6A modification of lncRNAs regulates cleavage, transport, stability, and degradation of lncRNAs themselves [59]. It also affects the biological function of cells such as cell proliferation and apoptosis, invasion and metastasis, cell stemness, and drug resistance in cancer, thereby enhancing the malignancy of cells and the difficulty of cancer treatment [60]. To date, several studies have enriched our understanding of the interaction between lncRNAs and m6A modifications. m6A can regulate lncRNA aberrant expression and lncRNA regulation of m6A modifications can alter normal biological processes. For example, m6A modification promotes the competitive endogenous RNA (ceRNA) of RHPN1-AS1 to act as a sponge for miR-596 by increasing the stability of the RHPN1-AS1 transcript or reducing RNA degradation, thereby increasing leucine zipper/EF hand-containing transmembrane-1 (LETM1) expression and activating the FAK/PI3K/Akt signaling pathway [61].

Meanwhile, lncRNA also influence the m6A machinery. The lncRNA lnc-H2AFV-1 upregulates the expression of intraflagellar transport (IFT) 80 by regulating METTL3/14 and FTO in head and neck squamous cell carcinoma (HNSCC), thereby promoting cell growth [62]. LINC00665 can modulate 11 mRNAs by regulating m6A enzymes YTHDF1, IGF2BP1, and IGF2BP2 in hepatocellular carcinoma (HCC) [63]. Furthermore, it is increasingly clear that m6A and lncRNAs may contribute to the clinical application of cancer therapy [64].

### m5C modification in lncRNA

RNA m5C methylation is methylation of the fifth C-position of RNA cytosine, which is a major post-transcriptional modification of RNA (Fig. 1) [65]. m5C modification has been shown to be widespread in mRNA and ncRNA, including lncRNA, tRNA, rRNA, and enhancer RNA (eRNA) [66]. Aberrant m5C methylation is associated with the onset and progression of certain cancers. In different RNAs, this modification has different functions. m5C regulates the structure and stability of tRNA. In rRNA, the loss of methylcytosine allows translation to be read by stop codons. The essential roles of m5C modification in mRNA are export and post-transcriptional regulation [67]. m5C methylation in eRNAs protects RNA from degradation [68]. RNA m5C methylation plays a crucial role in the regulation of RNA translation, stability, nuclear export, and other biological processes [69]. High-throughput sequencing analysis showed that m5C methylation sites were widely present in ncRNA [70, 71]. As the most abundant ncRNA species, a large number of m5C modifications are detected in lncRNAs [71].

In eukaryotes, C5 methylation of RNA cytosine is catalyzed by RNA methyltransferases (RNMTs). RNMTs belong to the DNA methyltransferase family (especially TRDMT1/DNMT2) or to the NSUN (NOL1/NOP2/sun domain) family (NSUN1/2/3/4/5/6/7) [72]. Most RNA methyltransferases have been shown to methylate rRNA (NSUN1/4/5), tRNA (NSUN2/3/5/6 and DNMT2), mitochondrial tRNA (NSUN2/3), and eRNA (NSUN7) [66, 73] as shown in Table 1. Extensive work has now shown that RNMT is aberrantly expressed and plays an important role in cancer development and pathogenesis [68, 74]. NSUN2 is involved in the m5C modification of many RNA, including mRNA, tRNA, lncRNA, rRNA, and miRNAs [71, 75–77]. NSUN1/2 was found to be a proliferation marker, highly expressed in various cancers, and associated with poor prognosis [78–80]. In mouse skin cells, NSUN2 was first identified as a target of MYC, and its deletion impairs MYC-dependent proliferation [81]. Afterward, recent studies have investigated its related potential pathways. For example, NSUN2-mediated aberrant m5C modification of lncRNA H19 is closely related to poor differentiation of HCC, and H19 lncRNA can specifically bind to the oncoprotein ras-GTPase-activating protein binding protein 1 (G3BP1) to further lead to MYC accumulation [32]. Furthermore, NSUN2-mediated lncRNA NMR promotes tumor progression by controlling the expression of important oncogenic drivers in esophageal squamous cell carcinoma, such as matrix metalloproteinase 10 (MMP10) and MMP3 [77]. Recently, it has also been reported that NSUN2 can promote the stability of carcinogenic mRNA of bladder cancer (BLCA) by depositing m5C [80].

For m5C demethylation, the ten-eleven translocation (TET) gene was initially thought to be a tumor suppressor gene [82], but it is subsequently thought to mediate oxidation to 5-hydroxymethyl, 5-formyl, and 5-carboxylcytosine (hm^5^C, f^5^C, and ca^5^C), then excise either f^5^C or ca^5^C. This may be induced by thymidine DNA glycosidase (TDG) in DNA [83]. TET protein-mediated DNA m5C demethylation was demonstrated in earlier studies [84]. After RNA is modified with m5C, proteins bind specifically to the modification sites, leading to subsequent modulation of biological processes. These specific proteins recognize m5C-containing oligonucleotides, including YTHDF2, Aly/REF export factor (ALYREF), and Y-box binding protein 1 (YBX1). Their specific effects in m5C are shown in Table 1. To date, the function of m5C modification in many types of RNA has been extensively studied. However, there are few studies on m5C in lncRNA. The study of m5C methylation in lncRNA is still in its initial stage [85].

### m7G modification in lncRNA

In recent years, with the gradual deepening understanding of m6A and m5C, research on m7G has gradually increased, making m7G modification the next research hotspot in RNA modification. m7G is a modification of RNA guanine (G) by adding methyl to its seventh N under the action of methyltransferase (Fig. 1) [86]. m7G modification is one of the most common base modifications in post-transcriptional regulation, which is widely distributed in tRNA, rRNA, lncRNA, and the 5 ‘cap region of eukaryotic mRNA. It plays an important role in maintaining RNA processing and metabolism, including mRNA transcription, mRNA translation, splicing, tRNA stability, nuclear processing, 18S rRNA maturation, and miRNA biosynthesis [87–90]. In humans, the METTL1/WD repeat domain 4 (WDR4) complex catalyzes N7-methylguanosine [91]. In this complex, METTL1 acts catalytically, while WDR4 stabilizes the role of METTL1. This complex extensively affects mRNA translation (Table 1) [92]. The research on m7G has gradually increased, and recent research has also found that m7G modifications regulate tumorigenesis and progression [93, 94]. Conceivably, targeting METTL1/WDR4-mediated m7G is a promising anticancer strategy. For example, METTL1-mediated modification of m7G tRNA upregulates epithelial growth factor receptor (EGFR)/EGF-containing fibulin extracellular matrix protein 1 (EFEMP1) expression, which ultimately promotes BLCA tumorigenesis [95]. Furthermore, METTL1 reduced the chemical resistance of colon cancer cells to cisplatin by upregulating miR-149-3p and targeting S100A4/p53 axis [96]. However, the specific mechanism of m7G modification in lncRNAs remains unclear. At the same time, methods to detect m7G modifications are constantly being updated, including m7G-Seq, m7G-MeRIP-Seq, and m7G-miCLIP-Seq technologies [97, 98].

## Functions of lncRNA-RNA modification in different types of cancer

### m6A-related lncRNA in cancer

The role of m6A modification associated with lncRNA in tumorigenesis and tumor suppression is being gradually explored by scientists. m6A modifications can induce structural changes in lncRNAs through writer or reader access to m6A sites. lncRNAs have also been shown to modulate downstream targets by modulating m6A. Here, we summarize the role of lncRNAs associated with m6A modifications in cancer.

### Lung cancer

**Fig. 2:**
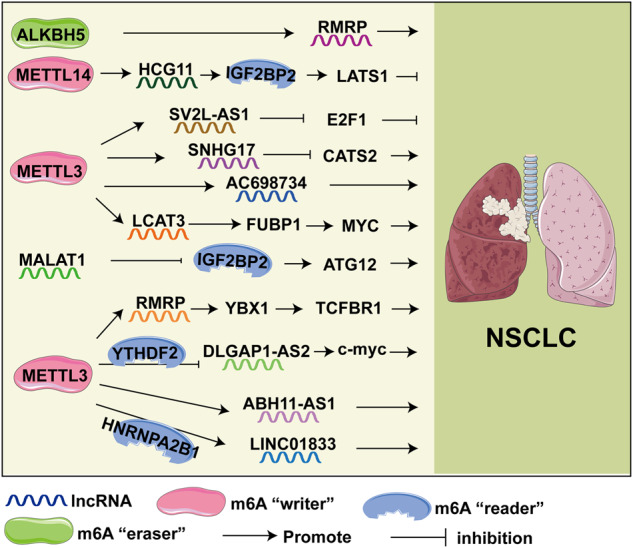
m6A-modified lncRNAs and signaling that participate in lung cancer.

Lung cancer is one of the most common malignancies with the highest mortality worldwide [99]. Lung cancer is divided into small-cell lung cancer (SCLC) and non-small-cell lung cancer (NSCLC) based on histological manifestations. NSCLC accounts for the vast majority of lung cancer cases [100]. Lung adenocarcinoma (LUAD) is the most common type of NSCLC. LncRNA and m6A modification are involved in the occurrence and development of lung cancer through various mechanisms (Fig. 2). Among the m6A writers, research on METTL3 and 14 is the most prevalent. Li et al. showed that LINC01833 m6A methylation triggered by METTL3 promoted NSCLC progression by regulating HNRNPA2B1 [101]. Another study reported that upregulation of LncRNA LCAT3 in LUAD has been attributed to METTL3-mediated m6A modification. LCAT3 activated MYC transcription by recruiting far upstream element binding protein 1 (FUBP1), thus promoting the progression of LUAD [102]. Moreover, the m6A transferase METTL3-induced lncRNA ABHD11-AS1 can promote NSCLC proliferation and Warburg effect [103]. LncRNA AC098934 promotes proliferation and invasion of LUAD cells by binding to METTL3 and m6A modification [104]. Besides, METTL3-mediated enhanced expression of the lncRNA SVIL antisense RNA 1 (SVIL-AS1) inhibited E2 promoter-binding factor 1 (E2F1), thereby inhibiting LUAD cell proliferation [105]. Zhang et al. demonstrated that METTL3-mediated lncRNA SNHG17 reduced gefitinib sensitivity in LUAD through epigenetic inhibition of large tumor suppressor kinase 2 (LATS2) expression [106].

m6A readers responsible for recognition in m6A modification also plays a role. METTL3-mediated lncRNA DLGAP1-AS2 interacts with YTHDF1 and enhances the stability of c-Myc mRNA through DLGAP1-AS2/YTHDF1/m6A/c-Myc mRNA. This promotes aerobic glycolysis and growth in NSCLC [107]. Work by Mao et al. revealed that lncRNA Human leukocyte antigen complex group 11 (HCG11) expression is downregulated in LUAD, which is regulated by METTL14-mediated m6A modification. The m6A modification of HCG11 promotes its binding to IGF2BP2. HCG11 acts as a tumor suppressor and suppresses tumor growth in LUAD by promoting Large Tumor Suppressor Kinase 1 (LATS1) [108]. IGF2BP2 can also upregulate the expression of autophagy-related (ATG) 12 by promoting the stability of MALAT1, which is conducive to the proliferation of NSCLC [109]. The lncRNA-RNA component of mitochondrial RNA processing endoribonuclease (RMRP) is highly upregulated in NSCLC. m6A-modified lncRNA RMRP stability promotes NSCLC proliferation and progression by regulating the transforming growth factor beta receptor 1 (TGFBR1)/SMAD2/SMAD3 pathway [110]. Furthermore, RMRP also promotes the development of LUAD, which is dependent on demethylation of ALKBH5 to upregulate RMRP expression. And ALKBH5 knockdown inhibited tumorigenesis of LUAD in *vitro* and in *vivo* [111].

### Liver cancer

**Fig. 3:**
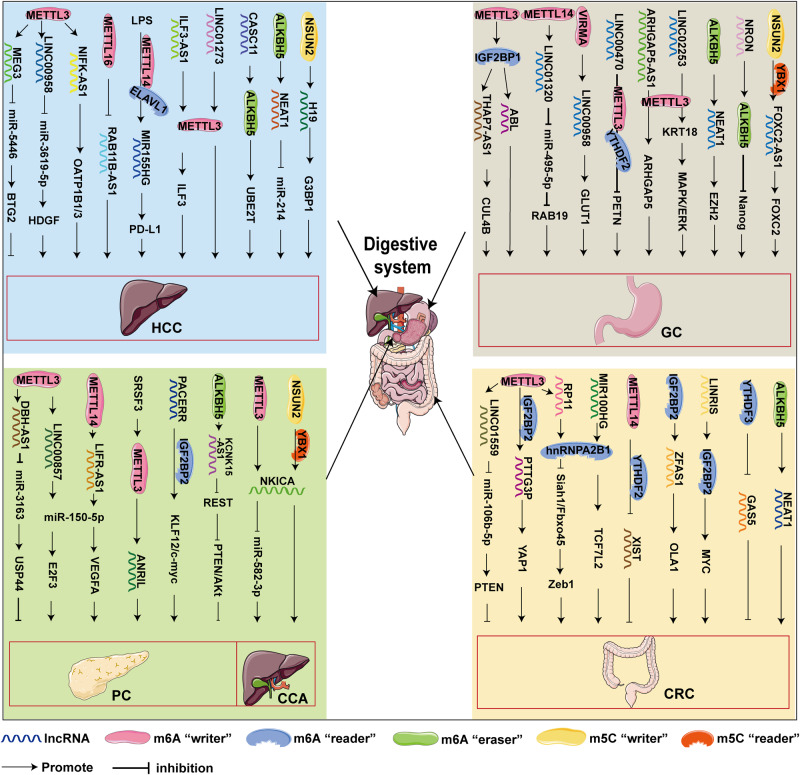
m6A and m5C-modified lncRNAs and modified elements that participate in digestive system cancers.

HCC is a common primary hepatocellular carcinoma with a relatively high mortality [112]. The main treatment options for HCC include surgical intervention, targeted therapy, liver transplantation, and immunotherapy. Although significant progress has made in the treatment of HCC in recent years, the high metastasis rate and postoperative recurrence rate still result in poor prognosis of HCC patients. Specifically, epigenetic mechanisms regulating the occurrence and progression of HCC are one of the main cause of this phenomenon [112]. LncRNAs modified by m6A regulators affect HCC proliferation, invasion and migration, adipogenesis, and drug resistance by regulating downstream targets (Fig. 3). Epigenetic studies have shown that METTL3-induced lncRNA MEG3 suppresses the proliferation, migration, and invasion of HCC cell through miR-544b/ BTG anti-proliferation factor 2 (BTG2) signaling [113]. Zuo et al. demonstrated that METTL3-mediated m6A modification leads to LINC00958 upregulation by stabilizing its RNA transcripts. Mechanistically, LINC00958 targets miR-3619-5p to upregulate the expression of hepatoma-derived growth factor (HDGF), thereby promoting HCC adipogenesis and progression [114]. Dai et al. found that METTL16 is upregulated in HCC and induces m6A modification of RAB11B-AS1, which reduces the stability of RAB11B-AS1 transcript, resulting in down-regulation of RAB11B-AS1 [115]. Peng et al. demonstrated that upregulation of METTL14 by lipopolysaccharide (LPS) promotes m6A methylation of the lncRNA MIR155HG, which relies on a “reader” protein ELAVL1 (also known as HuR)-dependent pathway to stabilize MIR155HG. LPS-induced MIR155HG upregulates PD-L1 expression and promotes immune escape in HCC [116].

As one of the demethylases, ALKBH5 can remove m6A modification on lncRNA to regulate the biological function of tumor. Yeermaike et al. showed that ALKBH5 could upregulate the expression of lncRNA NEAT1 by inhibiting m6A enrichment. NEAT1 promotes cell proliferation in HCC through sponge miR-214 [117]. On the other hand, lncRNAs can also target downstream targets by regulating m6A modification. LncRNA cancer susceptibility candidate 11 (CASC11) is upregulated in HCC and promotes HCC progression. Additionally, CASC11 regulates m6A modification of ubiquitin-conjugating enzyme E2 T (UBE2T) mRNA by binding to the RNA demethylase ALKBH5 [118]. LncRNA ILF3-AS1 increases the level of ILF3 m6A by recruiting METTL3, thereby stabilizing interleukin enhancer binding factor 3 (ILF3) mRNA to promote HCC progression [119].

In addition, lncRNA also regulates drug resistance of liver cancer cells through m6A modification. Sorafenib is the first-line drug approved for the treatment of advanced HCC. Nevertheless, the efficacy of sorafenib is greatly reduced due to the drug resistance of HCC. [120]. Studies have shown that LINC01273 confers sorafenib resistance in HCC by regulating METTL3 [121]. Moreover, Chen et al. found that METTL3 upregulated lncRNA NIFK-AS1 in HCC to promote disease progression and sorafenib resistance, and NIFK-AS1 made HCC cells resistant to sorafenib by downregulating the drug transporters organic anion transporting polypeptide (OATP)1B1 and OATP1B3 [122].

### Gastric cancer

Gastric cancer (GC) is a global health problem with a high mortality rate and a low survival rate [123, 124]. Most GC patients are diagnosed at an advanced stage of malignant proliferation and metastasis, and this late diagnosis often leads to a grim prognosis. Therefore, it is critical to identify new biomarkers and therapeutic targets to facilitate early diagnosis and precision treatment of GC. Many scholars have studied the interaction between lncRNA and m6A in GC, as well as the regulatory mechanism involving various genes and signaling pathways (Fig. 3). KIAA1429 accelerates aerobic glycolysis in GC through m6A-modified LINC00958 [125]. Furthermore, epigenetic studies showed that METTL14-mediated m6A modification promoted the expression of LINC01320. Overexpressed LINC01320 contributed to the aggressive phenotype of gastric cancer cells via regulating the miR-495-5p/RAB19 axis [126]. Liu et al. showed that METTL3-mediated m6A modification enhanced the expression of ThAP7-AS1, depending on the IGF2BP1-dependent pathway of the “reader” protein. ThAP7-AS1 promoted GC progression by improving CUL4B entry into the nucleus [127]. Studies have found that m6A-modified apoptotic protease-activating factor 1 (APAF1)-binding lncRNA (ABL) promotes tumor proliferation and drug resistance in GC by blocking apoptotic body assembly. IGF2BP1 combines with ABL and maintains its stability [128]. Yan et al. showed that LncRNA LINC00470 was upregulated in GC and promoted GC cell proliferation, migration, and invasion. LncRNA LINC00470 promotes PTEN mRNA decay via METTL3 in an m6A reader protein YTHDF2-dependent pathway [129]. Among m6A demethylases, ALKBH5 affects gastric cancer development by demethylating lncRNAs. Zhang et al. showed that ALKBH5-induced demethylation of lncRNA NEAT1 upregulated the expression of EZH2 (a subunit of the Polycomb repressive complex), thereby promoting the invasion and metastasis of GC [130]. The lncRNA NRON promotes GC proliferation by combining with ALKHB5 and mediating the decay of Nanog mRNA [131].

On the contrary, lncRNAs also regulate the m6A modification to affect the expression of the target. Hou et al. identified the lncRNA ARHGAP5-AS1 as an upregulated lncRNA in chemotherapy-resistant gastric cancer cells, whose knockdown reversed chemotherapy resistance. Interestingly, ARHGAP5-AS1 stabilizes ARHGAP5 mRNAs in cytoplasm by recruiting METTL3 [132]. Gao et al. demonstrated that LINC02253 increases m6A modification of keratin 18 (KRT18) mRNA by recruiting METTL3. KRT18 promotes the GC cell growth and metastasis by activating the MAPK/ERK signaling pathway [133].

### Pancreatic cancer

Pancreatic cancer (PC) is a very aggressive disease that is difficult to diagnose at an early stage. It progresses rapidly at a rate of about 1% per year [134, 135]. Pancreatic ductal adenocarcinoma (PDAC) is one of the most aggressive subtypes of PC, and late diagnosis and high heterogeneity are the greatest obstacles to its treatment. Despite ongoing efforts to improve the treatment of PDAC, the five-year survival rate for PC remains as low as 12% [136]. Therefore, there is an urgent need to discover novel biomarkers that facilitate early detection and improve treatment strategies. Abnormal m6A modification of lncRNA is currently found in PC tissues and cell lines (Fig. 3) [137, 138]. Chen et al. found that METTL14-modified LncRNA LIFR-AS1 promotes the progression of PC. LIFR-AS1 can directly interact with miR-150-5p, thereby indirectly upregulating the expression of vascular endothelial growth factor A (VEGFA) [139]. Meng et al. determined that METTL3-induced LINC00857 functions as a ceRNA to sponge miR-150-5p, leading to upregulation of its target E2F transcription factor 3 in PC cells and ultimately promoting tumorigenesis in PC [140]. During m6A methylation, IGF2BP2, which is responsible as “reader”, is also involved in PDAC progression. Liu et al. demonstrated that LncRNA-PACERR activates the KLF12/p-AKT/c-myc pathway by binding to miR-671-3p. Furthermore, LncRNA-PACERR bound to IGF2BP2 enhanced the stability of KLF transcription factor 12 (KLF12) and c-myc in the cytoplasm in an m6A-dependent manner. Both pathways induce pro-tumor macrophages in PDAC [141]. For m6A demethylases, one study found that ALKBH5 blocked m6A modification of KCNK15-AS1, enhancing the expression and stability of KCNK15-AS1 in PC cells. Furthermore, ALKBH5-mediated KCNK15-AS1 inhibits KCNK15 translation by binding to the KCNK15 5′UTR, and KCNK15-AS1 inhibits REST and inactivates the PTEN/AKT pathway to inhibit PC progression [142].

Gemcitabine-based chemotherapy remains an important option for all PC patients [143]. However, gemcitabine resistance can emerge within weeks of starting chemotherapy [144]. Gemcitabine resistance is one of the main reasons for clinical treatment failure of pancreatic cancer. Wang et al. demonstrated that upregulation of Serine/arginine-rich splicing factor 3 (SRSF3) is associated with gemcitabine resistance in PC. SRSF3 regulates splicing and m6A modification of lncRNA ANRIL in PC cells to promote gemcitabine resistance [145]. Ye et al. showed that an increased METTL3-mediated m6A modification of lncRNA DBH-AS1 can competitively bind to miR-3163 and upregulate ubiquitin-specific peptidase 44 (USP44), thereby inhibiting PC growth and gemcitabine resistance [146]. It can be concluded that lncRNA and m6A methylation are potential targets of chemotherapy resistance in pancreatic cancer. In addition, studies have shown that epigenetic inhibitors and gemcitabine have synergistic antitumor effects in PC cells [147]. Thus, dysregulation of lncRNA and m6A-modifying regulators in PC suggests their potential value as novel biomarkers in pancreatic cancer diagnosis and targeted therapy.

### Colorectal cancer

Colon cancer (CRC) is one of the most common malignant tumors in the world, with its incidence rate ranking third and mortality ranking second, which seriously reduces the quality of human life [148]. CRC is a malignant tumor that forms when abnormal cells in the colon or rectum divide uncontrollably. In view of the unclear symptoms of early CRC, nearly 60% are diagnosed at an advanced stage. The high mortality rate of CRC is mainly caused by tumor metastasis and recurrence, which are closely related to migration [149]. The lncRNAs associated with m6A modification have been found to play important roles in CRC (Fig. 3). Yang et al. showed that METTL14 inhibits the proliferation and metastasis of CRC by downregulating the oncogenic LncRNA XIST, and the m6A methylated XIST is recognized by the YTHDF2, thereby mediating the degradation of XIST [34]. Shi et al. showed that METTL3-mediated LINC01559 suppresses CRC progression by regulating the miR-106b-5p/PTEN axis [150]. METTL3 increases the expression of pituitary tumor-transforming 3, pseudogene (PTTG3P) by affecting its stability, while IGF2BP2 can recognize and bind to the PTTG3P m6A methylation status. PTTG3P promotes CRC progression by upregulating YAP1 [151].

In addition, as in other cancer types, the role of m6A reader proteins is also critical. Lu et al. showed that LncRNA ZFAS1 promotes the proliferation and apoptosis inhibition of CRC cells, which depends on the binding and recognition of IGF2BP2. ZFAS1 enhances Obg-like ATPase 1 (OLA1) activity and activates glycolysis in CRC cells by binding to the OBG-type domain of OLA1 [152]. Wang et al. found a highly expressed lncRNA LINRIS in CRC. LINRIS binds to the ubiquitination site of IGF2BP2, and this binding blocks the degradation of IGF2BP2 through the ubiquitination-autophagy pathway. Furthermore, MYC-mediated glycolysis is affected by the interaction between LINRIS and IGF2BP2 [153]. Interaction of lncRNA MIR100HG with hnRNPA2B1 promotes m6A-dependent stabilization of transcription factor 7 like 2 (TCF7L2) mRNA and colorectal cancer progression, which is also important for maintaining EMT-related cetuximab resistance [154]. Wu et al. demonstrated that METTL3-mediated lncRNA RP11 triggers the dissemination of CRC cell. RP11 binds to hnRNPA2B1 and down-regulates the mRNA expression of Siah1 and Fbxo45, thus stimulating the expression of Zeb1 after translation [35]. The decay of LncRNA GAS5 induced by YTHDF3 promotes CRC progression through YAP signal [155]. For m6A demethylases in lncRNAs, ALKBH5 was found to promote CRC progression by upregulating lncRNA NEAT1 expression through demethylation [156].

### Breast cancer

**Fig. 4:**
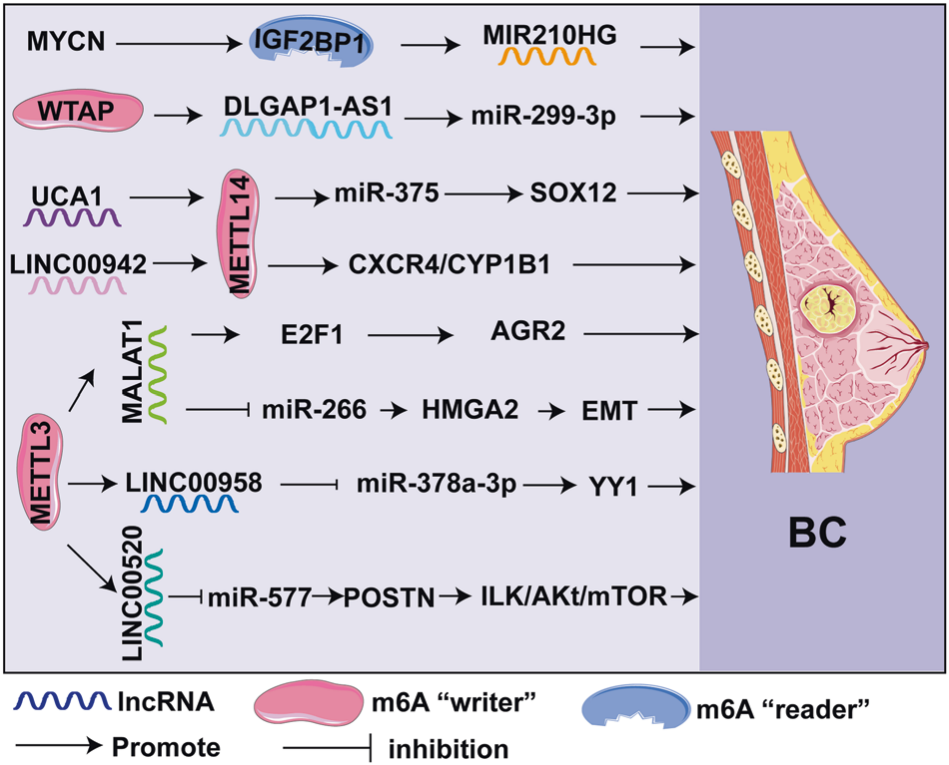
m6A-modified lncRNAs and modified elements that participate in breast cancer.

Breast cancer (BC) has been the leading cause of cancer death in women with a high degree of molecular heterogeneity [157]. Currently, surgical resection combined with radiotherapy and chemotherapy is still the most effective treatment for advanced BC, but the recurrence rate is still high [158]. With the advancement of technology, other novel therapies, such as molecular targeted therapy or immunotherapy, are increasingly used in BC. It is very important to study the molecular mechanism of BC metastasis to find new therapeutic targets, and new treatment strategies are urgently needed [159]. The interaction between lncRNA and m6A modification has also been investigated in BC (Fig. 4). It has been reported that m6A-modified upregulated LINC00520 as a ceRNA for miR-577 enhances POSTN levels, thereby activating the ILK/AKT/mTOR signaling pathway and promoting BC progression [160]. Sun et al. revealed that LINC00942 (LNC942) directly recruits METTL14 protein and stabilizes the expression of its target genes C-X-C motif chemokine receptor 4 (CXCR4) and CYP1B1 in BRCA initiation and progression through m6A methylation modification [161]. Rong et al. showed that upregulated LINC00958 promoted tumor progression in BC cells. Mechanistically, METTL3 caused the upregulation of LINC00958 by promoting the stability of its RNA transcripts. Furthermore, LINC00958 promotes YY1 transcription factor (YY1) as a competing endogenous RNA for miR-378a-3p [162]. However, METTL3-induced methylation of LINC00675 inhibited BC cell proliferation, invasion, and migration. Mechanistically, LINC00675 interacts with miR-513b-5p as a ceRNA and inhibits its expression [163]. The study found that the m6A-modified lncRNA MALAT1 promoted BC proliferation and adriamycin resistance. Zhao et al. showed that MALAT1 upregulated by METTL3 modification could enhance the expression of HMGA2 via sponge miR-26b. This promotes EMT, migration, and invasion of BC cells [164]. In addition, Li et al. also demonstrated that METTL3 modifies MALAT1 protein through m6A, recruits E2F1, and activates downstream AGR2 expression, thereby promoting adriamycin resistance in BC [165]. Meanwhile, Huang et al. demonstrated that WTAP binds to the m6A modification site of lncRNA DLGAP1 antisense RNA 1 (DLGAP1-AS1) and stimulates its stability, and enhances BC adriamycin resistance [166]. In addition, MYCN regulates lncRNA MIR210HG via IGF2BP1. The MYCN/IGF2BP1/MIR210HG axis promotes breast cancer progression [167]. In addition to this, LncRNA UCA1 can also regulate the m6A modification of miR-375 by METTL14 to promote the expression of SRY-box transcription factor 12 (SOX12) in BC [168].

### Head and neck cancer

**Fig. 5:**
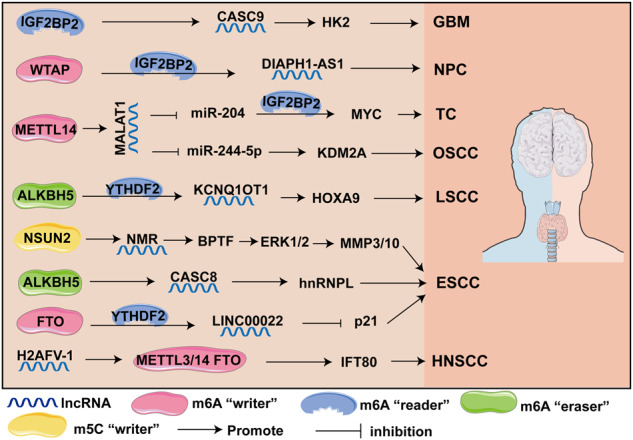
m6A and m5C-modified lncRNAs and modified elements that participate in head and neck cancer.

Head and neck cancers include neck tumors, ear, nose, and throat tumor, and oral and maxillofacial tumors. Head and neck cancers have many primary sites and pathological types. Thyroid cancer (TC) is the most common cancer in the neck. More than 90% of head and neck cancers are squamous cell carcinomas (head and neck cancers squamous cell carcinomas, HNSCC) [169]. The global incidence of HNSCC has increased markedly in the last 10 years, especially in women. HNSCC includes oral squamous cell carcinoma (OSCC), laryngeal squamous cell carcinoma (LSCC), and esophageal squamous cell carcinoma (ESCC) [170]. Li et al. showed that METTL14-mediated m6A modification increases the stability and expression of the lncRNA MALAT1, and the relative binding of MALAT1 to miR-224-5p promotes lysine demethylase 2A (KDM2A) transcription, thereby facilitating OSCC cell proliferation (Fig. 5) [33].

For m6A demethylase, FTO mediates m6A demethylation of LINC00022 and facilitates LINC00022 upregulation in a YTHDF2-dependent manner (Fig. 5). LINC00022 promoted the proliferation and cycle of ESCC cells by degrading p21 [171]. Li et al. showed that WTAP expression was apparently upregulated in NPC, and WTAP has enhanced the stability of DIAPH1-AS1 via m6A modification, which is also dependent on the recognition of IGF2BP2, ultimately facilitating NPC growth and metastasis [172]. Chen et al. showed that lncRNA H2AFV-1 increased the m6A modification of its downstream target IFT80 by upregulating METTL3/14 and downregulating FTO. This plays an important role in promoting HNSCC cell proliferation [62]. Furthermore, ALKBH5 mediates the hypomethylation and hyperexpression of lncRNA KCNQ1 overlapping transcript 1 (KCNQ1OT1), which depends on the recognition of YTHDF2. KCNQ1OT1 upregulates HOXA9 to promote the progression of LSCC cells [173]. In addition, ALKBH5-mediated m6A-induced lncRNA Cancer Susceptibility Candidate 8 (CASC8) also promoted ESCC proliferation and chemoresistance through upregulation of heterogeneous nuclear ribonucleoprotein L (hnRNPL) [174]. In TC, the lncRNA MALAT1 promotes the progression of TC cells by competitively binding to miR-204, upregulating IGF2BP2, and enhancing MYC expression [175].

### Other cancers

**Fig. 6:**
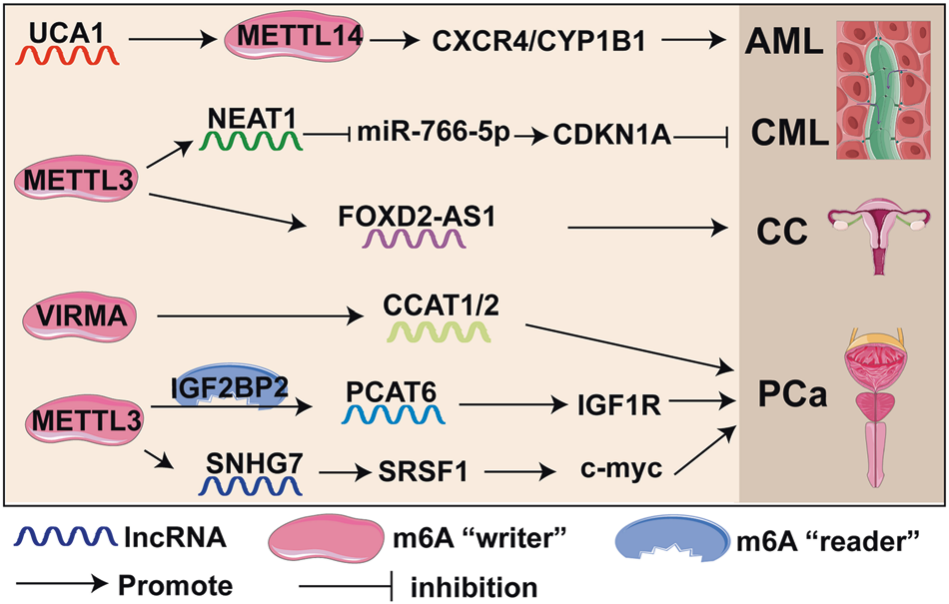
m6A-modified lncRNAs and modified elements that participate in other cancers.

In addition to the cancers mentioned above, m6A-related lncRNAs have been poorly studied in other cancers, such as prostate cancer (PCa), cervical cancer (CC), nasopharyngeal carcinoma (NPC), glioblastoma and leukemia (Fig. 6). For PCa, METTL3-mediated m6A modifies and stabilizes the lncRNA small nucleolar RNA host gene 7 (SNHG7), which regulates c-Myc by interacting with serine/arginine-rich splicing factor 1 (SRSF1), thereby accelerating glycolysis in PCa [176]. In bone metastasis-positive PCa, METTL3-mediated m6A modification promotes lncRNA PCAT6 upregulation in an IGF2BP2-dependent manner. PCAT6 promotes PCa bone metastasis by facilitating IGF1R mRNA [177]. In addition, VIRMA can also promote the expression of lncRNAs CCAT1 and CCAT2 in PCa dependent on m6A modification [178].

In osteosarcoma, the WTAP/FOXD2-AS1/m6A/FOXM1 axis promotes osteosarcoma progression. WTAP-mediated m6A modification of the lncRNA FOXD2-AS1 enhances the stability of FOXD2-AS1, thereby interacting with FOXM1 through m6A binding to increase FOXM1 expression [179]. For cervical cancer, METTL3/FOXD2-AS1 accelerates the cervical cancer progression via an m6A-dependent modality [180]. Liu et al. found that IGF2BP2-stabilized lncRNA CASC9 accelerates aerobic glycolysis in glioblastoma multiforme (GBM) by enhancing HK2 mRNA stability [181]. LncRNA UCA1 promotes acute myeloid leukemia (AML) progression by affecting the stability of METTL14 and upregulating the expression of CXCR4 and cytochrome P450 family 1 subfamily B member 1 (CYP1B1) [182]. Meanwhile, METTL3-modified lncRNA NEAT1 inhibits the progression of chronic myeloid leukemia (CML) by downregulating miR-766-5p targeting cyclin-dependent kinase inhibitor 1A (CDKN1A) [183].

These studies demonstrate that m6A is an important epitranscriptomic modification active in lncRNA-related cancer development and progression. Therefore, the role of lncRNA m6A modification in various cancers deserves further study to better understand the relevant mechanisms, which may provide new insights for early cancer diagnosis, outcome prediction, and cancer treatment strategies.

### m5C-related lncRNA in cancer

Recent studies on RNA methylation have mainly focused on the m6A modification of RNA, but there was little research on the modification of RNA m5C. NSUN2, as an RNA methyltransferase, plays an important role in various biological processes in cancer. In HCC, NSUN2-mediated aberrant m5C modification of H19 lncRNA can specifically bind to the oncoprotein G3BP1. This may be a new mechanism by which lncRNA H19 promotes tumorigenesis and development [32]. Li et al. discovered a novel NSUN2 methylated lncRNA NMR that promoted ESCC cell migration and invasion and increased drug resistance in ESCC cells. NMR binds to BPTF, potentially promoting the expression of MMP3 and MMP10 through the ERK1/2 pathway [77]. Zhen et al. found that NF-kappa B interacting lncRNA (NKILA) was upregulated in Cholangiocarcinoma (CCA). NKILA was modified by m5C mediated by NSUN2 and m6A mediated by METTL3. NKILA enhanced the expression of YAP1 by inhibiting miR-582-3p [184]. Furthermore, FOXC2-AS1, which is highly expressed in GC, recruits NSUN2 to FOXC2 mRNA, increases its m5C level and combines with YBX1. FOXC2-AS1 acts as an oncogenic lncRNA in an m5c-dependent manner by stabilizing FOXC2 mRNA, which may provide a new therapeutic target for GC [185]. m5C-related lncRNAs have been found to play important roles in regulating the tumor-immune microenvironment in uterine corpus endometrial carcinoma (UCEC) and BLCA [85, 186]. Figures 3 and 5 also summarizes the role of m5C modification in the regulation of tumor-associated lncRNAs.

Recently, High-throughput sequencing data showed that m5C-related lncRNAs were associated with tumor-immune cell infiltration and could be used as potential therapeutic targets for a variety of tumors [187, 188]. He et al. screened and validated six m5C-related lncRNAs in stomach adenocarcinoma (STAD) using bioinformatics and statistical analysis. HAGLR and AC009948.1 are risk genes, while AC005586.1, AL590666.2, AP001271.1, and IPO5P1 are protected genes. According to gene set enrichment analysis, these lncRNAs are associated with multiple immune-related pathways and are involved in immune cell infiltration [189]. Song et al. comprehensively analyzed the cross-talk between 141 m6A- and m5C-related lncRNAs in CRC, indicating that they have potential impacts on tumor immunity, microenvironment and clinicopathological features, such as ALMS1-IT1, NNT-AS1, SNHG22, STAM-AS1, NR2F1-AS1, LINC00628 and CASC2, etc [190]. Zhang et al. first explored m5C-associated lncRNAs in lower-grade gliomas (LGG), resulting in prognostic biomarkers ZBTB20-AS4, LINC00265, GDNF-AS1, and CIRBP-AS1 [191]. In addition, five lncRNAs related to m5C (AL031985.3, AL928654.1, ELNF1-AS1, MKLN1-AS and NRAV) have been found to be upregulated in HCC, and they have potential functions in tumor prognosis, immune cell infiltration, and drug sensitivity [192]. However, these studies lack further research on how lncRNAs interact with m5C.

### m7G-related lncRNA in cancer

Epigenetic modifications of lncRNAs such as m6A and m5C have been proven to be associated with the occurrence and progression of various cancers [65, 193]. Unfortunately, whether and how m7G modification participates in cancer progression by regulating lncRNAs remains unclear. Similar to m6A and m5C, m7G has recently been shown to play an important role in cancer. For example, METTL1 is associated with advanced tumor stage, vascular invasion, and poor prognosis in HCC patients, and promotes tumor progression by increasing the translation of target mRNA by promoting m7G modification of tRNA [93, 194]. In recent years, a large number of bioinformatics studies have focused on m7G modifications associated with lncRNAs. Many scholars have evaluated the prognosis and tumor immunity of many cancers by constructing m7G-related lncRNA risk models, which prompts further research on m7G modification mechanisms in lncRNAs. Yang et al. predicted novel m7G-related lncRNAs for colon cancer prognosis and tumor-immune microenvironment, including 8 lncRNAs, namely MCM3AP-AS1, ELFN1-AS1, PCAT6, GABPB1-AS1, GS1-124K5.4, SNHG7, ZEB1-AS1, and C1RL-AS1 [195]. In addition, another study reported 9 m7G-related lncRNAs in LIHC which were indicative of prognosis. They show potential value in predicting prognosis, drug sensitivity, and immunotherapy response in LIHC patients [196]. Seventeen m7G-related lncRNAs have also been reported in CRC, which can be used to predict prognosis in the clinical setting and to determine whether the tumor is cold or hot in CRC to improve the individualization of treatment [197]. In addition, there are similar studies in ESCC, UCEC, CM, and LUAD [198–201] as shown in Table 2.

**Table 2:** Prognosis value of m7G-related lncRNAs in cancer.

Cancer	lncRNA		Effect	Reference
	protective factors	risk factors		
CRC	MCM3AP-AS1, ELFN1-AS1, C1RL-AS1, GABPB1-AS1, ZEB1-AS1, SNHG7, PCAT6, and GS1-124K5.4		Prognosis, tumor-immune microenvironment	[195]
	AL137782.1, AC012313.5, AL031985.3, AC007728.3 and AC099850.4	AC012313.5, AC133540.1, AC083900.1, AL137782.1, LINC00702, AP001619.1, ALMS1-IT1, AC013652.1, LINC02550, AC008760.1, AP006621.2 and FGF14-AS2	Prognosis, diagnosis of CRC cold and hot tumors	[197]
	AP02305AC 5, ZKSCAN2-DT, AC073896.3, AL512306.2, AL354993.2, AC003101.2, AL137782.1, AC005014.2, AC092944.1, U91328.1, AC004540.2, AC104819.3, RNF216P1, LINC00997, AC004846.1, AC078820.1, LINC02593, AC139720.2, ZEB1-AS1 and AL513550.1		Prognosis, tumor-immune infiltration	[237]
LIHC	SOCS2-AS1, RP5-1171I10.5, RP11-588H23.3, RP11-10A14.3 and NAV2-AS4	RP11-43F13.3, RP11-95O2.5, RP11-519G16.5 and RP11-874J12.4	Prognosis, drug sensitivity, immunotherapy response	[196]
ESCC	AC025754.2、 AL4 51165.2 and AL513550.1	HAND2-AS1、SNHG7、SRP14-AS1和AC007566.1	Prognostic, tumor microenvironment heterogenicity	[198]
UCEC	AC010378.1、DNAJC3-DT and AC139887.1	NBAT1, LEMD1-AS1, LINC00662, AC004951.1, AC019080.5, AC011466.1 and AL031667.3	Prognostic and immune function analysis	[199]
CM	AATBC, MCCC1-AS1, AC018529.2, AC099811.3, AC015911.3 and AC125807.2		Immune functions, immune cell infiltration, immune components, tumor microenvironment	[200]
LUAD	LINC01352, SALRNA1, AP000695.1, AC026355.2, AC018647.1 and AL355472.3		Immune cell infiltration, drug sensitivity	[201]

*UCEC* Uterine corpus endometrial carcinoma, *CM* Cutaneous melanoma.

## Future perspectives and conclusions

RNA modification, especially m6A modification, plays an important biological role in various types of cancer, and the development of targeted drugs based on m6A modification has become a promising treatment strategy. For example, the first m6A inhibitor (STM-2457) targeting METTL3 has entered phase I clinical trials in 2022. Both in *vivo* and in *vitro* experiments showed that the drug can inhibit the proliferation of AML [202]. Cheng et al. developed two potent FTO inhibitors, FB23 and FB23-2, which directly bind FTO and selectively block its m6A demethylase activity, significantly inhibiting the proliferation of AML cell lines and primary maternal AML cells [203]. Later, they discovered that FTO inhibitors CS1 and CS2 can inhibit the self-renewal of cancer stem cells and enhance T cell toxicity [204]. In addition, for m5C methylase, azacitidine, and decitabine are cytidine analogs that inhibit any m5C methylase and have been approved for clinical use in hematological malignancies [205]. Abnormally expressed m6A-related lncRNAs were recently discovered in the peripheral blood of HCC patients, suggesting that m6A-modified lncRNAs have good clinical application prospects as biomarkers [206]. Over the past few years, the development of lncRNA therapeutics have been witnessed [207], and the field of RNA-modifying proteins as drug targets is expanding [208]. Unfortunately, the dysregulated expression of lncRNAs associated with RNA modifications in cancer has not yet been exploited in clinical settings. Detailed studies on the distribution and function of lncRNA-related RNA modifications and their interactions with upstream and/or downstream targets will contribute to understanding the regulatory network of multiple genes and pathways in cancer. Therefore, it is of great significance and value to elucidate the mechanism of lncRNA-related RNA modifications in tumor development, screen and explore potential targets, and validate in preclinical studies to help establish new diagnosis and treatment strategies.

This review summarizes the role of m6A, m5C, and m7G modifications of lncRNAs in cancer, but further studies of lncRNA-related m5C and m7G are needed, as well as studies focusing on less-studied proteins, such as m6A-related RBM15/ 15B, CBLL1, ABCF1, eIF3 and FMR1, etc. Further investigation of the interactions between different RNA modifications on tumorigenesis is required, such as the interaction between m6A and m5C. However, due to the complex molecular mechanism of RNA modification in lncRNAs in cancer, it is still challenging to apply its findings to clinical practice. But this does not prevent us from developing small molecule modulators targeting RNA modification sites and RNA modification enzymes, which will provide a targeted approach to cancer treatment. Although the strategies associated with RNA modification in lncRNAs are promising, extensive research is needed to depict the regulatory network of RNA modification in lncRNAs in cancer.
